# Association of Human Papilloma Virus Infection and Oral Squamous Cell Carcinoma in Bangladesh

**DOI:** 10.3329/jhpn.v31i1.14750

**Published:** 2013-03

**Authors:** Mahmuda Akhter, Liaquat Ali, Zahid Hassan, Imran Khan

**Affiliations:** ^1^Department of Oral and Maxillofacial Surgery, Faculty of Dentistry, Bangabandhu Sheikh Mujib Medical University, Dhaka, Bangladesh;; ^2^Bangladesh Institute of Health Science, Darus Salam, Mirpur, Dhaka, Bangladesh;; ^3^Department of Physiology and Molecular Biology, BIRDEM, 122 Kazi Nazrul Islam Avenue, Dhaka, Bangladesh

**Keywords:** Human papilloma virus (HPV), HPV type 16, HPV type 18, Oral cancer, Polymerase chain reaction, Bangladesh

## Abstract

Oral squamous cell carcinoma is the sixth most common malignancy worldwide. In Bangladesh, it comprises 20% of the whole body malignancies. Several studies found that 15% to 25% of oropharyngeal cancer cases are associated with human papilloma virus (HPV). This study is done to find the association of human papilloma virus subtypes, particularly HPV type 16 and HPV type 18, with the oral squamous cell carcinoma in Bangladeshi patients. In total, 34 diagnosed patients of oral squamous cell carcinoma were included in the study. Extracted DNA from the cancerous tissues was checked for PCR reaction to detect the subtypes of human papilloma virus. Data of the present study suggest that oral squamous cell carcinoma are almost absent in Bangladeshi patients with human papilloma virus, particularly HPV 16 and 18.

## INTRODUCTION

Oral squamous cell carcinoma (OSCC) is the sixth most common malignancy worldwide, with major incidence in western countries as well as India and Southeast Asia (1,2). Risk factors involved in the pathogenesis of oral cancer include infectious agents, low intake of fruits and vegetables, betel quid-chewing, as well as smoking and alcohol consumption (1,2). Recently, the possible association of oral pre-cancer lesions with cancer has also been suggested by the discovery of human papilloma virus (HPV) (3,4), like the high-risk HPV subtypes HPV 16 and HPV 18.

Detection of HPV may be dependent on several factors, e.g. molecular technique employed to detect the DNA materials, treatment of the sample material, ethnic and geographical differences, and the anatomic site of the lesion (5). Since 0% to 64% of individuals with carcinoma of the tonsil have detectable HPV, some researchers have investigated the prevalence of HPV in the oral cavity mucosa (6,7).

In western countries, researchers have used retrospective case studies to investigate the possible link between the high-risk HPV strains and oral squamous cell carcinoma (OSCC). A recent study (8) showed that, in the majority of the oral cancer cases with high risk of HPV, have HPV DNA. In an article published in the Journal of the National Cancer Institute in 2000, Gillison and colleagues examined tumours in 253 patients aged 17 to 91 years with newly-diagnosed or recurring squamous cell cancers of the head and neck (9). They found association of HPV in 25% of the tumours. Among those, more than 90% of the HPV-positive tumours contained HPV 16. People with HPV-positive tumours were 59% less likely to die of their cancer compared to patients with HPV-negative tumours. Co-factors that further increase the risk of invasive cancer among HPV DNA-positive women include older age, long-term use of oral contraceptives, high parity, smoking, and HIV infection (10,11).

In Bangladesh, no studies on the association of HPV subtypes with oral squamous cell carcinoma were published. This study is expected to assess whether Bangladeshi people possess the risk of having oral squamous cell carcinoma of HPV origin as reported in the literature of the western countries.

## MATERIALS AND METHODS

### Study design

It is a case series study done during the period of January 2009 to December 2009 in the Department of Oral and Maxillofacial Surgery, Bangabandhu Sheikh Mujib Medical University (BSMMU) and in the laboratory of the Department of Physiology and Molecular Biology of BIRDEM.

### Subjects

Clinically-diagnosed 45 subjects were enrolled in the study, irrespective of race, religion, socioeconomic status, and geographic distribution. Among them, 34 cases were diagnosed by biopsy as oral squamous cell carcinoma.

### Collection of tissue samples

After infiltration with local anaesthesia, tissue was incised, with all aseptic precaution, in such a manner that both normal and pathological tissues are included.

### DNA extraction

Extraction of integrated DNA from the sample tissue was done, using GenElute DNA extraction kit (QIAGEN, USA). Tissue sample was minced and placed in a micro-centrifuge tube to which protienase K was added. Contents of the tube with tissue buffer solution were incubated. Cell lysis buffer solution was readded to the tube and again incubated. After cooling the tube contents to room temperature, absolute ethanol was added and centrifuged. The column formed due to centrifuge was washed with buffer 1 solution, re-centrfuged, and again washed with the buffer solution 2. Then the resultant contents were taken in a DNA-collection tube to which DNA AE buffer was added and incubated, and the centrifuging was repeated. GenElute DNA kit was used for checking the yield and stored for future use.

### Polymerase chain reaction (PCR) for HPV

All samples were subjected to PCR, using primers specific for consensus sequence spanning the E6 open reading frame of high-risk HPV type 16, 18, 31, and 33.

PCR was carried out in 15 μL reaction volume which contained :

- 5 μL DNA solution
- 1.5 mM MgCl_2_
- 200 μM dNTPs
- 500 μM of each primer
- 1 unit of HotStart Taq DNA polymerase (Qiagen, USA)

The PCR conditions were as follows: initial incubation at 94 °C for 15 min followed by 35 cycles of reaction with step of denaturation at 95 °C for 45 seconds, annealing at 57 °C for 45 seconds and elongation at 72 °C for 45 seconds, and the 35th cycle was followed by a step of final elongation at 72 °C for 10 min.

PCR product was checked for amplification in a 3% agarose gel stained with ethidium bromide. The optimum size of the product was ascertained comparing it with 100 bp DNA ladder. Positive samples were subjected to PCR, using HPV 16 and 18 type-specific primers. PCR product-size for HPV 16 is 238 bp and for HPV 18 is 268 bp.

For HPV 16, PCR was carried out using the primer set:

Forward: 5’-ATTAGTGAGTATAGACATTA-3’

Reverse: 5’-GGCTTTTGACAGTTAATACA-3’.

Reaction was carried out in 15 μL reaction volume, and the conditions were :

Step of denaturation at 95 °C for 30 sec, annealing at 52 °C for 45 sec. and elongation at 72 °C for 45 sec for 35 cycles with an initial incubation at 95 °C for 15 min, and a step of final elongation at 72 °C for 10 min.

For HPV 18, PCR was carried out using the primer set:

Forward: 5’-ACTATGGCGCGCTTTGAGGA-3’

Reverse: 5’-GGTTTCTGGCACCGCAGGCA-3’.

Reaction was carried out in 15 μL reaction volume, and the conditions were as follows:

Step of denaturation at 95 °C for 30 sec, annealing at 64 °C for 45 sec, and elongation at 72 °C for 45 sec for 35 cycles with an initial incubation at 95 °C for 15 min, and a final elongation at 72 °C for 10 min.

For both reactions, PCR product was checked for amplification in 3% agarose gel stained with ethidium bromide.

## RESULTS

**Figure 1.**
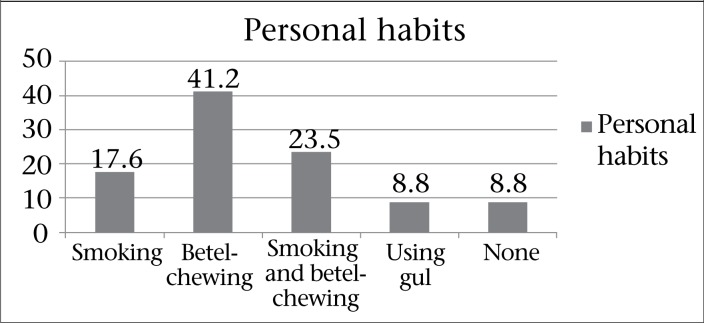
Distribution of the respondents by personal habit

Figure 1 shows the distribution of personal habits of the squamous cell carcinoma patients. Among the squamous cell carcinoma patients, 17.6% were smokers, 41.2% were betel-chewer, 23.5% were habituated to both smoking and betel-chewing, and 8.8% were used to taking gul. Rest 8.8% did not consume any form of tobacco or betel.

Among the 34 respondents, those who were diagnosed as quamous cell carcinoma patients, only 1 (2.9%) was in histological Grade 1, and the majority 23 (67.6%) were in Grade 2, and rest 10 were in Grade 3 (Table 1).

**T1able 1.** Distribution of the respondents by grading of the tumour

Grading of tumour	Frequency	Percentage
Grade 1	1	2.9
Grade 2	23	67.6
Grade 3	10	29.4
Total	34	100.0

Only one specimen was found to have HPV, out of 34 oral squamous cell carcinoma patients (Table 2).

**T1able 2.** Detection of HPV

HPV status	Frequency	Percentage
HPV present	01	3.0
HPV absent	33	97.0
Total	34	100

**Figure 2.**
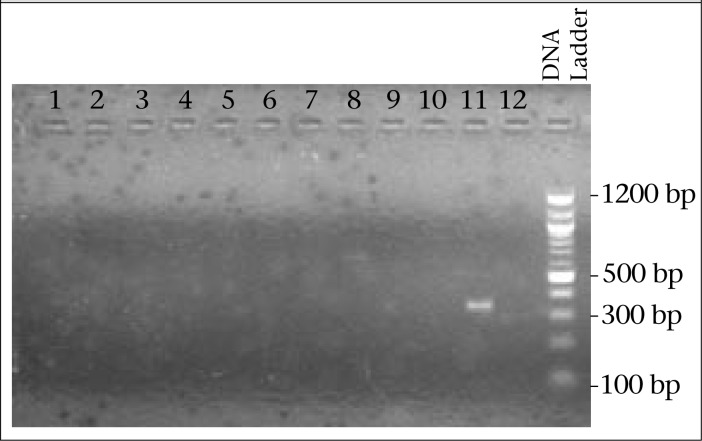
Image of agarose gel (3%) electrophoresis

In Figure 2, it is shown that positive sample-size is 340 bp, checked by 3% agarose gel electrophoresis, which is not specific for 16 or 18 type of HPV.

DNA sequencing is a standard procedure to confirm DNA product. The specific primer set did not produce any product of optimum size; so, absence of HPV DNA was inferred.

## DISCUSSION

The human papilloma viruses are a family of icosahedral, non-enveloped viruses with a circular, double-stranded DNA genome, which has special affinity for epithelial cells. So far, more than 120 different types of HPV have been isolated (1,2). Low-risk HPVs, such as HPV 6 and 11, induce benign hyperproliferations of the epithelium, such as papillomas, or warts. By contrast, high-risk oncogenic types (e.g. HPV 16, 18, 31, 33, and 35) are defined by there strong epidemiologic association with cervical cancers (12). Studies showed that HPV-related oral cavity lesions had higher prevalence, particularly among the younger age-group (13), although they may not develop oral cancers. HPV has also been linked to several head and neck carcinomas (5).

High-risk human papilloma viruses type 16 and 18, as aetiological agents of anogenital carcinomas, have been firmly established in the literature (14). Because of the morphological similarities (15) and epitheliotrophic nature of HPV, a link between OSCC and HPV seemed logical and has been the focus of numerous studies.

A predilection of HPV for certain, especially non-keratinized anatomical sites in the oropharynx, was confirmed when HPV 16-related DNA sequences were detected in 16%, 51%, 60%, and 13% of SCC of the tongue, tonsil, Waldeyer's ring, and pharynx respectively (7,16,17).

Estimates of the prevalence of oral HPV in normal mucosa, pre-malignant lesions, and OSCC are highly variable, and comprehensive reviews have reported results that varied from 0% to 100%. These results seemed to depend on the sampling methods, patient's profile (18,19), detection methods used (20), and the anatomical location of the tumours (9).

The major aim of this study was to see the possibility of having any evidence for HPV association as a risk factor of OSCC in our community, and if so, to find out the presence of type 16 and type 18 particularly.

The prevalence of HPV in oral cavity lesions varies widely, even when the detection of the virus is done, using polymerase chain reaction (PCR). Shroyer *et al.* (4) reported 10% prevalence while Watts *et al.* (3) reported 90% prevalence in carcinomas of the oral cavity. Also, there are other studies that show great variability in the prevalence of HPVs from 1% to 81.1% (21-24), although PCR-based assays were employed to detect HPV DNA in all the studies.

In the present study, only one specimen was found to have HPV out of 34 oral squamous cell carcinoma patients. The crude prevalence estimated is 3% (2.94). This study provides some evidence as mentioned.

It has been noted that there is decrease in the HPV PCR positivity with increasing distance from the site of the tumour. Part of the tumour where the DNA copy number is mostly available is designated as focal point of HPV infection (25). So, site for collecting tissue biopsy may be the one of the factor for variable prevalence.

Despite the mismatches at the base nucleotide inserted in the outer reverse primer region, the primers are able to amplify HPV. This is probably due to the low annealing temperature which allows for maximum amplification efficiency and for mismatches between the primers and template DNA. The primers have also shown that these are able to detect a broad range of mucosal HPV types and are, therefore, likely to be able to amplify novel mucosal HPV types (25). Also, there are limitations with various PCR methods.

Infection of the mucosa with HPV may increase the susceptibility of the epithelium to subsequent chemical carcinogenesis, and the nature of these co-carcinogens may vary from region to region and with the social and dietary habits of various people. This variation in both incidence of HPV infection of the oral mucosa and the nature of the chemicals that act as promoters, initiators, or as simple co-carcinogens of the SCC, may be important in explaining the wide variability of HPV detection in various head and neck carcinomas around the world (25). This variability may be noted for the Bangladeshi people who like highly-spiced food, have different dietary habits, high incidence of betelnut intake, and also take high quantity of vegetables grown with chemical fertilizers.

Polymerase chain reaction (PCR) is the sensitive method available for detecting HPV in the tissues. However, a problem with the PCR-based detection is the possibility of false positivity, due to carry-over of the product and contamination of paraffin-embedded tissue during routine processing (6). Also, due to diversity of the papilloma virus genome, there is no universal consensus primer set to detect all types of HPV (6).

In this study, we tried to find any association of HPV with oral squamous cell carcinoma among Bangladeshi population. The association between HPV and head/neck cancers are well-established, although the aetiological factors are still unknown. It is seen that HPV has predilection for primary tonsillar carcinomas but is rare for the pharynx or the hypopharyx (6). As this study was conducted on a small sample-size and the laboratory kit we used may differ in primer sets from the other kits, we recommend that this be converted to a cross-sectional study with a large sample-size and with different PCR kits and multiple primer sets. It is highly recommended that real-time PCR be used for more sensitive detection, keeping in mind that HPV association with oral squamous cell carcinomas also may have anatomical site preferences.
